# One size does not fit all: uncontrolled T2 inflammation on tezepelumab—a case series

**DOI:** 10.1186/s13223-026-01037-x

**Published:** 2026-04-24

**Authors:** Arianne Tardif, Marie-Eve Boulay, Andréanne Côté, Krystelle Godbout

**Affiliations:** 1 Institut universitaire de cardiologie et de pneumologie de Québec – Université Laval, 2725, Chemin Ste-Foy, Quebec City, QC G1V 4G5 Canada; 2 Faculty of Medicine, Université Laval, Quebec City, QC Canada

**Keywords:** Severe asthma, T2 inflammation, Anti-TSLP, Evasion

## Abstract

**Background:**

Tezepelumab is the most recent human monoclonal antibody in the therapeutic arsenal of patients with severe uncontrolled asthma. It neutralizes thymic stromal lymphopoietin (TSLP). Phase 2 and phase 3 studies showed a significant reduction in asthma exacerbations in the severe asthma population, with the greatest effects seen in patients with a type 2 (T2) phenotype. TSLP is in close relation with the epithelium which is believed to be the starting point of the inflammatory cascade. Hence, blocking TSLP is expected to fully control type 2 inflammation and its actors such as interleukin 4 (IL-4), IL-13, IL-5 and eosinophils. This case report describes asthma patient cases with uncontrolled T2 inflammation on tezepelumab and explores the potential evasion mechanisms of TSLP blockage.

**Cases presentation:**

We report four patients who developed symptomatic uncontrolled T2 inflammation following the initiation to tezepelumab. All cases were biologic-experienced and achieved best asthma control with benralizumab. Several hypotheses have been proposed to explain this phenomenon. One possibility is an immune-driven process within the spectrum of eosinophilic granulomatosis with polyangiitis (EGPA). In EGPA, eosinophils act as immunoregulatory cells and have a critical role in promoting the T2 response, which truly sanction the disease as an eosinophilic immune dysfunction disorder. With such process, targeting upstream signals, such as TSLP may prove ineffective in controlling the self-sustained eosinophilic process. Additionally, local airway immunity, even in the absence of EGPA, could influence the suboptimal response to anti-TSLP therapy by exacerbating the in situ T2 response.

**Conclusion:**

Tezepelumab is not the optimal treatment for all asthma patients requiring biologic therapy. A non-epithelial derived source of inflammation might explain the suboptimal response. These cases stress the importance of phenotyping and individualisation of therapy as essential steps of the severe asthma care.

## Background

Severe asthma care has been revolutionized in the past two decades by the development of monoclonal antibodies [1]. While these therapies reduce exacerbations [2–5], asthma remission can only be achieved in a subset of patients [6, 7]. This unmet need emphasizes that, despite the major strides made in our understanding of severe asthma and its pathophysiology, many unknowns remain.

In recent years, there has been a growing interest in the role of airway epithelium as an asthma driver and many now believe that most asthma cases originate from an epithelial dysfunction [8, 9]. Far from being a passive barrier, the epithelium also regulates mucociliary clearance and is a key player in the activation of the immune system. In response to different environmental aggressors, it releases epithelial cytokines known as alarmins (interleukin IL-25, IL-33 and thymic stromal lymphopoietin (TSLP)) that in turns interact with naïve T cells and innate lymphoid cells (ILC) to further promulgate type 2 and non-T2 inflammatory processes [10]. Therefore, in theory, inactivating alarmins has the potential to completely suppress the asthmatic inflammatory cascade, particularly of type 2 (T2) origin. Evidence has indeed shown that tezepelumab, a biologic directed against TSLP, decreases exacerbations in T2 and non-T2 asthmatics and reduces tissue eosinophils, an end-product and central cell of T2 inflammation [3, 11, 12].

We report here 4 patients treated with tezepelumab who displayed uncontrolled symptomatic eosinophilic inflammation while on the drug, leading to its discontinuation. These cases might provide insights on a common underlying mechanism of disease that is resistant to tezepelumab. As per GINA 2025, we defined T2 inflammation as a blood eosinophil count ≥ 150 cell/uL, FeNO ≥ 20 ppb, and sputum eosinophils ≥ 2%.

The Institut Universitaire de Cardiologie et de Pneumologie de Québec-Université Laval (IUCPQ-UL) is a tertiary center with a multidisciplinary severe asthma clinic.

## Cases presentation

### Case 1

**Fig. 1:**
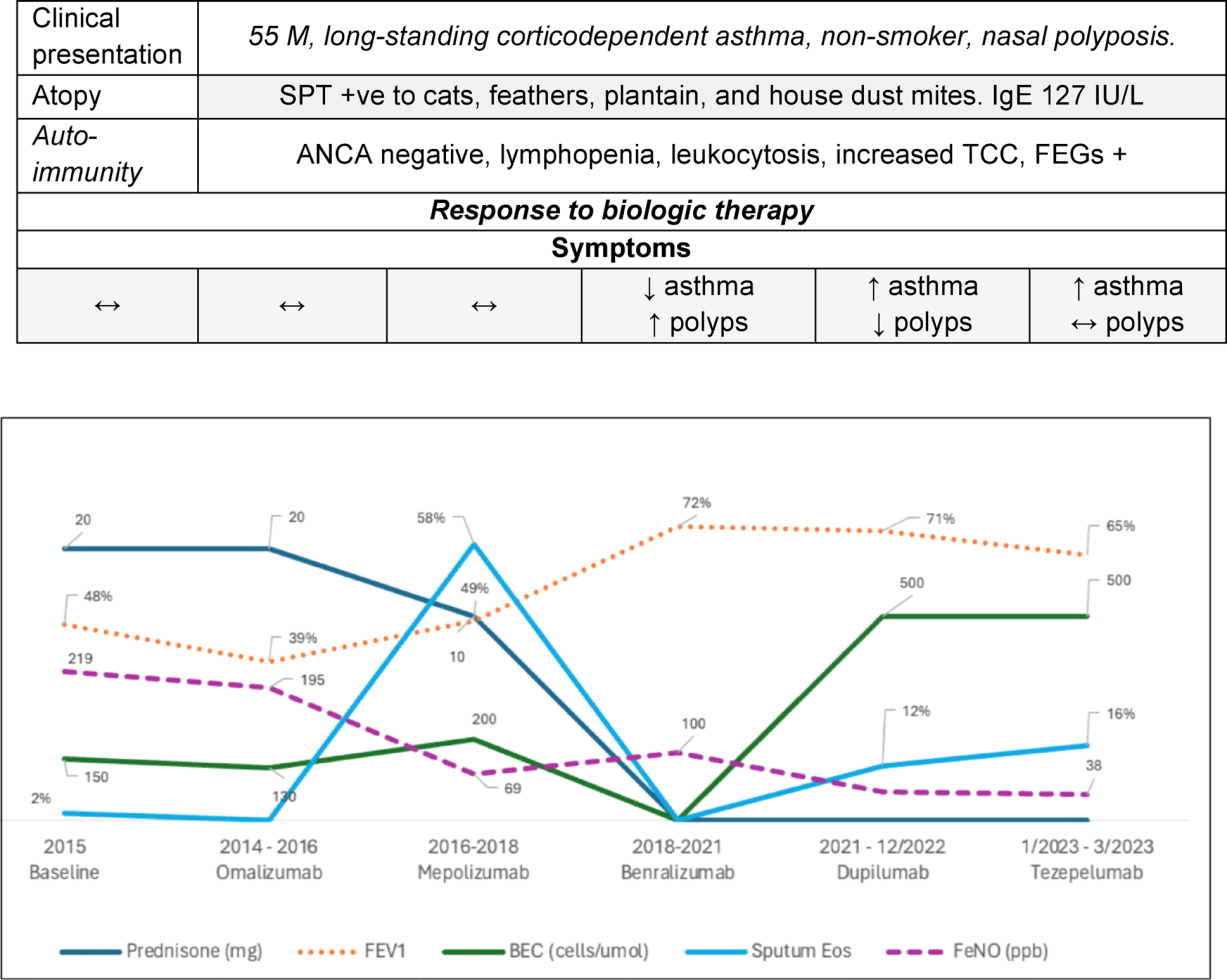
Case 1 overview of presentation and biomarkers responses to different biologics agents. Baseline values represent the first available results for each test obtained at our institution. For each biologic therapy, the values shown correspond to the last measurement recorded prior to switching. Lymphopenia is defined as blood absolute lymphocyte count < 1.5 × 10^9^/L on 3 occasions. Leukocytosis is defined as absolute white blood cell count > 11 × 10^9^/L. Increased TCC is defined as TCC > 10 × 10^6^ cell/g. *BEC* Blood eosinophil count, *FEG* Free eosinophil granulates, *SPT* Skin prick test, *TCC* Total cell count

This patient is a 55-year-old male diagnosed with severe asthma since his early twenties and comorbid nasal polyps with past surgery. Despite being a never smoker, he had chronic obstruction. At medical takeover, the patient was dependent of oral prednisone (20 mg daily). Despite being treated with oral corticosteroids (OCS), high dose inhaled corticosteroids (ICS), long-acting beta agonist (LABA) and Montelukast, the patient remained symptomatic. Initial workup on prednisone showed no residual eosinophilic inflammation but his fractional exhaled nitric oxide (FeNO) was markedly elevated (Fig. 1). No persistent polyp was found on ear, nose and throat (ENT) evaluation.

Omalizumab was first introduced but did not provide improvement or reduction in OCS. The patient was therefore switched to Mepolizumab, again with no clinical benefit. Severe eosinophilic airway inflammation was unmasked when attempting to wean OCS on Mepolizumab and the patient was switched to benralizumab. Benralizumab provided marked clinical improvement and prednisone was completely weaned with no asthma worsening. FeNO remained high with no clinical consequences. The nasal polyps however recurred upon prednisone cessation, causing burdensome symptoms. For this reason, benralizumab was switched for dupilumab. The polyposis improved quickly, and both conditions were initially well controlled. After 3 months on dupilumab, a clear deterioration in his asthma control was witnessed along with airway eosinophilia recurrence, probably precipitated by the benralizumab washout.

The patient was switched to tezepelumab in the hope of controlling both his asthma and nasal polyps. He reported a clinical deterioration quickly after the first injection. The forced expiratory volume in one second (FEV_1_) decreased and T2 inflammation flared up after 2.5 months on tezeplumab. The patient was put back on benralizumab and his asthma symptoms abated, concomitantly with a complete control of his eosinophilic airway inflammation.

### Case 2

**Fig. 2:**
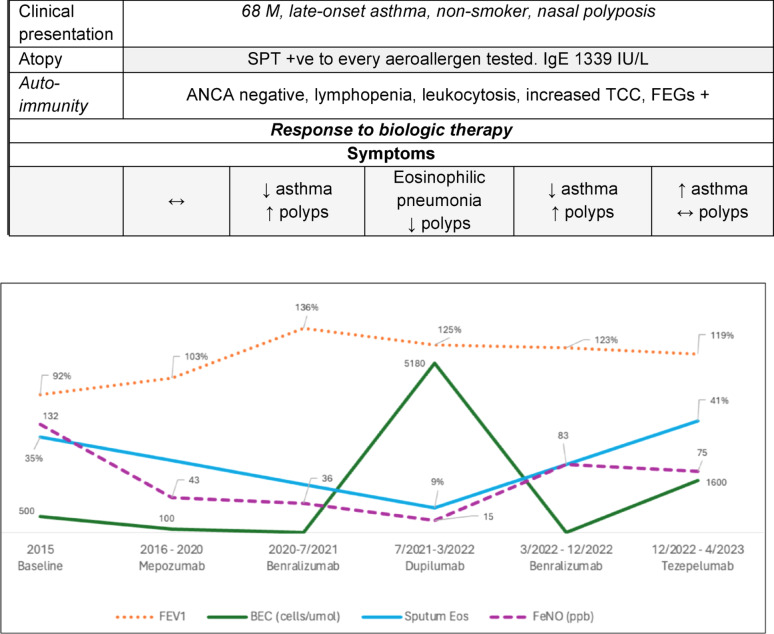
Case 2 overview of presentation and biomarkers responses to different biologics agents. Baseline values represent the first available results for each test obtained at our institution. For each biologic therapy, the values shown correspond to the last measurement recorded prior to switching. Lymphopenia is defined as blood absolute lymphocyte count < 1.5 × 10^9^/L on 3 occasions. Leukocytosis is defined as absolute white blood cell count > 11 × 10^9^/L. Increased TCC is defined as TCC > 10 × 10^6^ cell/g. *BEC* Blood eosinophil count, *FEG* Free eosinophil granulates, *SPT* Skin prick test, *TCC* Total cell count

This patient is a 68-year-old male with an asthma diagnosis at age 56 and comorbid nasal polyps requiring surgery. Despite high dose ICS/LABA, Tiotropium and Montelukast, he remained symptomatic and suffered 3 exacerbations in the year prior to biologic initiation. Baseline phenotyping shown in Fig. 2 demonstrated significant T2 inflammation especially on induced sputum where many free eosinophil granules (FEGs) were also seen.

Mepolizumab was introduced with an initial good response on his symptoms and exacerbations. They worsened two years later with a constantly suboptimal and obstructive FEV_1_ and two exacerbations. While no eosinophilic inflammation could be uncovered, his FeNO remained elevated. A switch to benralizumab was attempted. After initiation of benralizumab, the patient felt significantly better and his FEV_1_ reached a personal best. The FeNO level remained elevated with no clinical consequence. His nasal polyps, however, worsened and impacted his quality of life. For this reason, he was switched to dupilumab. On this drug, the patient had a better control of his polyps, but his asthma control worsened with a resurgence of eosinophilic inflammation (Fig. 2). He was ultimately diagnosed with an eosinophilic pneumonia. It justified the medication cessation and re-introduction of benralizumab with a good response.

As the polyps recurred on benralizumab, tezepelumab was attempted in the hope of treating adequately both conditions. Although polyps improved, asthma control worsened and the T2 inflammation recurred 4 months after switching. The patient was put back on benralizumab, bringing a perfect asthma control.

### Case 3

**Fig. 3:**
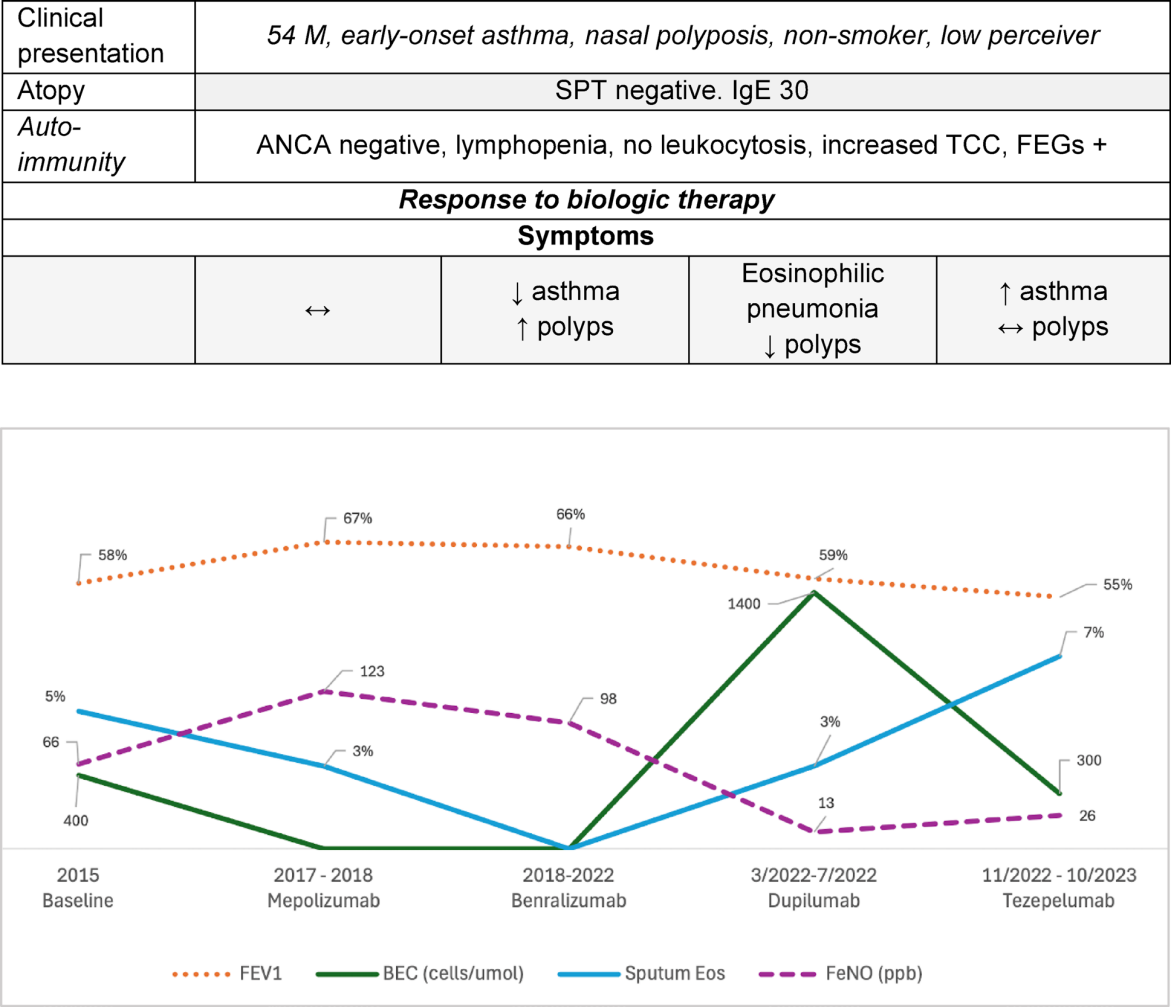
Case 3 overview of presentation and biomarkers responses to different biologics agents. Baseline values represent the first available results for each test obtained at our institution. For each biologic therapy, the values shown correspond to the last measurement recorded prior to switching. Lymphopenia is defined as blood absolute lymphocyte count < 1.5 × 10^9^/L on 3 occasions. Leukocytosis is defined as absolute white blood cell count > 11 × 10^9^/L. Increased TCC is defined as TCC > 10 × 10^6^ cell/g. *BEC* Blood eosinophil count, *FEG* Free eosinophil granulates, *SPT* Skin prick test, *TCC* Total cell count

This patient is a 54-year-old male, lifelong non-smoker, with asthma since his early twenties and comorbid nasal polyps with past surgery. He’s had bronchial thermoplasty in the setting of a clinical trial. On high dose ICS/LABA, his FEV_1_ was 58% predicted with a significant bronchodilator reversibility. Initial phenotyping showed evidence of non-atopic T2 inflammation and airway neutrophilia (Fig. 3). No remaining polyps were found on ENT assessment.

Mepolizumab was introduced because of persistently suboptimal lung function and 2 exacerbations. It did not provide improvement in his symptoms nor lung function, but he stopped exacerbating. A switch to benralizumab was attempted, the response to which was deemed unclear after a year, justifying its discontinuation. Upon cessation, exacerbations recurred together with a worsening in symptoms and recurrence of T2 inflammation (Fig. 3). Benralizumab was resumed, which abated the patient’s symptoms and exacerbations. After 2 years on benralizumab, his nasal polyps worsened and impacted his quality of life. A switch to dupilumab was performed which provided great improvement in his nasal symptoms but a worsening of his respiratory condition. A diagnosis of eosinophilic pneumonia with concomitant infection was made. OCS was initiated, dupilumab stopped and benralizumab was resumed. The patient reached his personal best FEV_1_. During the prednisone weaning, the symptoms and FEV_1_ worsened with high FeNO (53 ppb) without blood eosinophils.

Tezepelumab was prescribed to improve his lung function and symptoms without the need of OCS. He initially improved but asthma control worsened, and eosinophilic inflammation recurred eleven months after the switch from benralizumab (Fig. 3). Tezepelumab was stopped, and the patient eventually returned on benralizumab.

### Case 4

**Fig. 4:**
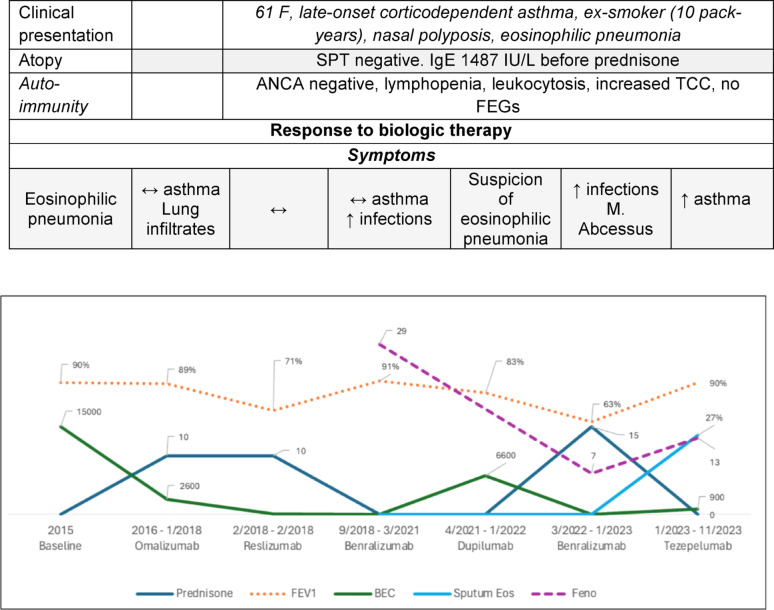
Case 4 overview of presentation and biomarkers responses to different biologics agents. Baseline values represent the first available results for each test. For each biologic therapy, the values shown correspond to the last measurement recorded prior to switching. Lymphopenia is defined as blood absolute lymphocyte count < 1.5 × 10^9^/L on 3 occasions. Leukocytosis is defined as absolute white blood cell count > 11 × 10^9^/L. Increased TCC is defined as TCC > 10 × 10^6^ cell/g. *BEC* Blood eosinophil count, *FEG* Free eosinophil granulates, *SPT* Skin prick test, *TCC* Total cell count

This is a 61-year-old female with an asthma diagnosis at age 49, comorbid nasal polyps requiring surgery and rheumatoid arthritis treated with leflunomide. Seven years before the referral, she had a diagnosis of idiopathic eosinophilic pneumonia with a negative extensive workup for secondary causes. She responded well to OCS, but her asthma control worsened when OCS were discontinued without relapse of her eosinophilic pneumonia (Fig. 4).

A presumptive diagnosis of allergic bronchopulmonary pulmonary aspergillosis was initially made by an allergist based on an intradermal reaction to *Aspergillus fumigatus* and high IgE before OCS initiation. The IgE levels had however since decreased and the cutaneous reaction and serum precipitins to *Aspergillus* were negative. She was treated with itraconazole and omalizumab. Asthma symptoms improved and OCS were successfully weaned. Six months after stopping the OCS, T2 inflammation and lung infiltrates recurred with no increase in total IgE levels. Prednisone was reintroduced and omalizumab switched to reslizumab. It did not provide clinical improvement nor OCS reduction. reslizumab was switched to benralizumab which provided immediate improvement in the patient symptoms and increased her FEV_1_ to her personal best. OCS was weaned. After one year on benralizumab, the patient reported end-of-dose symptomatic deterioration and suffered from an exacerbation which warranted a switch to dupilumab. She worsened on this medication with a recurrence of her eosinophilic pneumonia. OCS was reintroduced, benralizumab resumed, and she was referred to our asthma clinic.

The initial workup at the clinic, while on prednisone 15 mg due to recent presumed eosinophilic pneumonia, and benralizumab, showed isolated airway neutrophilia with cultures positive for *Nocardia nova*, *Aspergillus fumigatus* and group B *Streptococcus*. Bronchiectasis with signs of chronic infection were found on the chest CT. Treatment for nocardiosis was initiated but her condition failed to improve. She remained OCS-dependent and suffered recurrent pulmonary infections with resistant pathogens. benralizumab was doubted as a contributing factor and the patient was switched to tezepelumab with the aim to reduce respiratory infections. In the meantime, sputum cultures came back positive for *Mycobacterium abscessus* and treatment was initiated. With the anti-mycobacterial drugs and tezepelumab, asthma symptoms and FEV_1_ improved, and prednisone was weaned. Symptomatic eosinophilic inflammation appeared 2 months after OCS discontinuation (10 months after tezepelumab initiation). She was put back on benralizumab and T2 inflammation became undetectable and her FEV_1_ optimal without OCS.

## Discussion

### Review of the cases

Our four cases displayed common clinical features and response to therapy that may suggest a shared mechanism of disease. Upon phenotyping, all were found to have severe eosinophilic inflammation, 2 of which were OCS-dependent. Despite their eosinophilic phenotype, the response to mepolizumab or reslizumab, was disappointing, with minimal improvement in their asthma control, whereas benralizumab showed best asthma control.

In severe asthma, the more profound depletion of airway eosinophils achieved with benralizumab has been associated with improved clinical responses in patients with persistent eosinophilic inflammation despite treatment with mepolizumab or reslizumab [13]. In real-world studies, switching from mepolizumab to benralizumab has also been shown to reduce residual exacerbations [14], likely reflecting more complete eosinophil depletion, particularly as half the exacerbations occurring on mepolizumab are eosinophilic in nature [15]. In EGPA, while benralizumab was demonstrated to be non-inferior to mepolizumab for the primary outcome [16], two notable differences emerged: benralizumab led to a higher proportion of patients who discontinued OCS and greater asthma remission rates [17]. Taken together these findings suggest that, in selected patients like our four reported cases, benralizumab offer clinical advantages through more complete eosinophil depletion in both asthma and EGPA.

All patients also shared nasal polyps as a comorbidity and the uncontrolled nasal symptoms on benralizumab prompted a switch to dupilumab in 3 patients. dupilumab was however ineffective in controlling or worsened the eosinophilic inflammation in our patients, leading to clinical deterioration. Three of our 4 patients experienced an eosinophilic pneumonia while on dupilumab, that manifested after the benralizumab washout, although it was a recurrence in one patient.

All our patients noticed a clinical deterioration with recurrence of eosinophilic inflammation after switching to tezepelumab. This is surprising considering that previous studies have shown a reduction in blood and sputum eosinophils with tezepelumab [18]. This inconsistency in response to tezepelumab attest to the heterogeneity of asthma, even within a single phenotype. It also shows that targeting the alarmins alone might not be the best treatment option for all. We presented here patients that somehow evaded TSLP blockage. This arise several questions including if eosinophils can be recruited and activated by other mechanisms than the ones currently promoted in the literature.

Half the patients in our case series were OCS-dependent before initiating biologics. Tezepelumab failed to demonstrate OCS sparing effect in the SOURCE trial [19]. The poor response of our OCS-dependent patients to tezepelumab suggests that the drug may truly be ineffective for OCS-sparing, again raising the possibility of a TSLP-independent pathophysiological mechanism in those patients.

### Hypothetical mechanisms

A simple mechanism that could explain suboptimal response to tezepelumab would be the development of neutralizing anti-drug antibodies. These are however quite rare events with tezepelumab, being reported in 2/1059 patients in NAVIGATOR trial [3]. The similarities in the clinical pictures of our four cases hint towards a common endotypic pathway or others mechanism that eludes TSLP blockage. Therefore, although the presence of anti-drug antibodies has not been assessed, we believe other processes can better explain their poor response to tezepelumab.

All our patients had nasal polyposis and displayed severe eosinophilic airway inflammation, two for which chronic OCS was needed. The marked eosinophilic burden, which failed mepolizumab or reslizumab, may rise suspicion for EGPA. However, none of the patients had evidence of vasculitis or active end-organ involvement at the time of biologic initiation. EGPA is a rare and heterogeneous disorder that carries significant diagnostic challenges. As it lacks a specific biomarker, the diagnosis lies on the demonstration of vasculitis or evidence of tissue eosinophils infiltration with organ damage [20]. Nevertheless, it is well accepted that a prodromal phase characterized by severe asthma and rhinosinusitis with or without NP exists and can last for years. Asthma treatment with OCS or biologics may mask or prevent the development of other disease features, contributing to the diagnostic challenge. Is it therefore possible that the cases we presented lie in the spectrum of EGPA, at the prodromal phase, and share its distinct pathophysiological mechanisms, unresponsive to anti-TSLP.

Just as asthma, the pathophysiology of EGPA is complex and incompletely understood, at is roots a profound immunological dysregulation. EGPA is considered a T2 disorder, confirmed by the elevated levels of T2 cytokines (IL-4, IL-5, IL-10 and IL-13) in both in vivo and in vitro models [21, 22]. Eosinophils, the end products of the T2 pathway, are found in profuse number in serum and tissues and act as key effector cells through the cytotoxic and procoagulant effects of their released granules [23–25]. However, eosinophils in EGPA also act as immunoregulatory cells and have a critical role in promoting the T2 response. They are the main source of IL-25, a potent eosinophilotropic cytokine that induces their own proliferation and T2 response, thereby maintaining a vicious cycle [26]. Eosinophils can therefore be considered the sole perpetrators of non-vasculitic manifestations of EGPA, which truly sanction the disease as an eosinophilic immune dysfunction disorder. With such process, targeting upstream signals, such as TSLP is unlikely to control the self-sustained process. A more direct eosinophil target with anti-IL5 or anti-IL5R is expected to yield better results, and this has been shown in clinical trials with mepolizumab and benralizumab [27, 28] and has been observed in our cases, supporting an immune-driven disease in the spectrum of EGPA.

The presence of ANCA in the sputum, in the absence of serum detection has recently been uncovered in patients EGPA and is believed to be pathogenic in the autoinflammatory cascade [29]. Sputum ANCAs induce eosinophils cytolysis creating in turn a meshwork of DNA and eosinophils granules called eosinophils extracellular traps (EETs). EET production (EETosis) is a highly immunogenic event that activates epithelial cells to release IL-6 and IL-8, stimulates IL-5 and IL-13 secretion from ILC and leads to more autoantibody production triggering further EETosis and perpetuating the cycle [30, 31]. It is associated with severe asthmatic manifestations in EGPA patients [29]. This in situ response is again independent of the epithelium-driven asthmatic inflammatory cascade and may form another immune-driven mechanism by which our cases were tezepelumab-resistant. The presence of FEGs in 3 of our 4 cases certainly support ongoing EETosis.

Local airway autoimmunity has been observed in severe asthma in the absence of EGPA. In a study by Salter and colleagues, 61% of severe asthma patient had evidence of an in situ polyclonal autoimmune event defined as 2 or more autoantibodies detectable in the sputum [32]. This process is independent of a systemic etiology, attesting that this breach in immune tolerance is indeed an in-situ process. The presence of multiple airway autoantibodies was linked to blood lymphopenia, free eosinophil granules (FEGs) on sputum (evidence of EETosis) and recurrent infections (defined as leukocytosis, neutrophilia of increase total cell counts on sputum analysis), the latter probably mediated by macrophage dysfunction [32, 33]. In retrospective, three of the 4 cases displayed this characteristic triad. It can therefore be speculated that airway autoimmunity participated in the anti-TSLP suboptimal response of our patients. Indeed, through EETosis induction and immune-complex activation of C1q, autoantibodies heighten in situ T2 response, a process theoretically independent of TSLP [34]. Autoantibody-triggered eosinophil cytolysis were also found to be steroid resistant [35] and their presence is unaffected by current therapy, including biologics [32] which may explain the poor response to tezepelumab.

## Conclusion

Symptomatic uncontrolled T2 inflammation on tezepelumab happens, attesting that, the one size fits all approach should not be applied to biologics selection. Phenotyping is still a necessary step that cannot be overlooked in severe asthma patients. The poor control of eosinophilic inflammation with an anti-alarmin therapy suggests that the underlying process is not epithelium-driven but rather immune-driven. Whether it represents EGPA in its prodromal phase or airway autoimmunity remains to be further investigated but a self-sustained local airway inflammatory process is likely at play. Sputum proteomics and autoantibodies assessment may yield better discriminative properties in the phenotyping process, refining the underlying mechanism to better guide therapy.

## Data Availability

No datasets were generated or analysed during the current study.

## Abbreviations

ANCA: Antineutrophil cytoplasmic antibodies
BEC: Blood eosinophil count
CT: Computerized tomography
EET: Eosinophils extracellular traps
EGPA: Eosinophilic granulomatosis with polyangiitis
ENT: Ear nose and throat
FEG: Free eosinophil granules
FEV1: Forced expiratory volume in one second
FeNO: Fractional exhaled nitrous oxide
HASM: Hypereosinophilic asthma with systemic manifestation
IUCPQ-UL: Institut Universitaire de Cardiologie et de Pneumologie de Québec-Université Laval
ICS: Inhaled corticosteroids
Ig: Immunoglobulin
IL: Interleukin
ILC: innate lymphoid cells
IU: International unit
LABA: Long-acting beta-agonists
NP: Nasal polyps
OCS: Oral corticosteroids
SPT: Skin prick test
T2: Type 2
TSLP: Thymic stromal lymphopoietin
